# The use of micro-computed tomography in the diagnosis of dental and oral disease in rabbits

**DOI:** 10.1186/s12917-014-0209-4

**Published:** 2014-09-05

**Authors:** Hiroshi Sasai, Hiroyuki Iwai, Daisuke Fujita, Eiko Seto, Yuki Izumi

**Affiliations:** Kitasuma Animal Hospital, Hyougo, 9-5-8 Yokoo Suma-ward, Kobe City, Hyogo 654-0131 Japan

**Keywords:** Computed tomography, Micro-CT, Dentistry, Rabbit

## Abstract

**Background:**

The aim of this study was to investigate the use of a newly developed micro-computed tomography (micro-CT) system for the diagnosis of oral pathologies in small animals, using the rabbit as a model. The diagnosis of dental diseases in rabbits is usually based on oral endoscopy and radiographic imaging, but detailed pathological diagnosis using these methods is frequently difficult. Micro-CT was used in this study to address this challenge.

**Results:**

This study was conducted using 50 privately owned rabbits, presented to our hospital due to loss of appetite or difficulty feeding. Image recording times were 18 s in normal mode and 120 s in fine mode. The animals were maintained in the required position for scanning via the administration of sedatives. Micro-CT captured with a slice thickness of 60–120 mm has excellent spatial resolution, and is suitable for the clinical diagnosis of dental diseases in rabbits weighing 1–3 kg.

**Conclusions:**

Micro-CT can yield more detailed data than radiography or conventional CT. This study determined that this novel imaging modality can be utilized for the accurate assessment of dental and oral diseases in rabbits.

## Background

Dental diseases and associated complications, including apical abscesses of the maxilla and mandible, are common in pet rabbits. These conditions can become quite severe, leading to a loss of the ability to feed, and even death if left untreated [1]. In rabbits, the diagnosis of dental diseases is based on clinical signs and symptoms, oral endoscopy, and radiography of the head [1,2]. Radiography is a useful diagnostic modality in rabbits suspected of having dental diseases. Due to superimposition, making an accurate diagnosis requires multiple radiographic projections [3].

Micro-computed tomography (micro-CT) has remarkable space resolution, and a shorter capture time than cone beam computed tomography (CBCT). By coupling it with highly developed image reconstruction technology, it is possible to actively conduct imaging examinations in laboratory animals and other small animals [4]. CBCT has been used in the image-based evaluation of medical conditions in dogs and cats [5], and in anatomical studies on rabbits and guinea pigs [3,6-8]. In most cases, its clinical applications have been limited by low resolution, or an excessively long image processing time [7-9].

Radiographic evaluation of the skull, teeth, and soft tissues of the head requires capturing multiple images from various angles. Endoscopy is limited to the crowns. Not all types of CT imaging require the use of various positions, as is the case with radiography. The shortest possible implementation time is 18 s, and the time needed for image reconstruction is 1 minute. Thus, image reconstruction can be performed while scanning, reducing the overall time required for scanning, image processing, and performing the assessments required for diagnosis.

## Methods

### Rabbits

With each owner’s consent, micro-CT was used while performing clinical examinations and prognostic evaluations of 50 rabbits (mean age 4.7 ± 2.6 years), which were presented to our clinic due to loss of appetite or difficulty feeding, resulting from dental diseases. The study included 36 males and 14 females, and the breeds presented included Netherland dwarfs (*n* = 8), Holland lop-eared rabbits (*n* = 8), mini-rabbits (*n* = 10), and other breeds (*n* = 24). The diagnoses included mandibular abscesses and osteolysis (*n* = 24), maxillary tooth root growth lesions (*n* = 11), excessively long molar apices (*n* = 12), excessively long molar crowns (*n* = 25), excessively long incisors (*n* = 5), periapical infection of incisors (*n* = 4), maxillary abscesses (*n* = 4), and orbital abscesses (*n* = 3).

### CT device and image acquisition conditions

An X-ray tube with 5-μm focus and a 2-dimensional (2D) flat panel detector was used to acquire 512 slice images, in a cylindrical range with a diameter of 60 × 60 mm or 73 × 60 mm in 18 s with one rotation of the rotating arm. In whole-body scan mode, 512 × 2 slice images were acquired in a cylindrical range of 60 × 120 mm or 73 × 120 mm. These were used as appropriate for the size of the rabbit’s skull.

**Figure 1:**
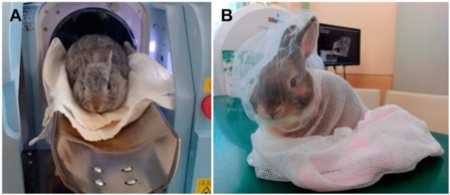
**Micro-CT scanner illustrating the gantry and non-anesthetized rabbit positioning. (A)** Non-anesthetized rabbit placed under a cone beam computed tomography (CBCT) scanner. **(B)** An animal-positioning cover with netting was created to maintain unsedated animals in position during scans.

The device used in this study was configured with a shield structure, so that an additional lead shield chamber was not required to protect the veterinarians operating the device from X-ray exposure. Its compact size allows it to be used in standard-sized examination rooms, and its diameter of 190 mm can accommodate animals weighing up to 3 kg inside the gantry. Depending on the rabbit’s level of activity and symptoms, CT imaging was conducted either with or without the administration of anesthesia (Figure 1). Many rabbits become docile when covered with a cloth or net, and by taking advantage of this behavior, we were able to diagnose 70% of the rabbits via 18 s of imaging without the use of anesthesia. Anesthesia was used if the rabbit was active, or if detailed diagnosis required imaging in fine mode, which required 120 s of scanning. Imaging was conducted either with a tube voltage of 90 kV and a tube current of 160 mA, or tube voltage of 70 kV and a tube current of 80 μA. The face spot size was set to a minimum of 5 μm and a maximum of 27 μm. An amorphous silicon detector was used, and image reconstruction was performed using the Feldkamp-Davis-Kress algorithm. Measurement of body fat, and synchronization with respiration were also conducted.

CT images (minimum: 10 mm pixel) were visualized with the number of pixels set to 512 × 512 × 512 (voxel = 10 × 10 × 10 μm) where the imaging time was 18 s, or 512 × 512 × 512 (voxel = 100 × 100 × 100 μm) where the imaging time was 120 s. Volume data was obtained from a cylindrical area 73 mm in diameter and 60–120 mm based on the height of the animal. For animals scanned under anesthesia, the scanning plane was vertical because complete positioning was possible. The acquisition of micro-CT images does not require a vertical plane however, so we were able to perform the procedure without anesthesia in cases where the animal’s behavior was conducive to doing so and the scanning time was 18 s.

We modified a previously reported approach to micro-CT used in laboratory guinea pigs and rats [10-14], to perform the diagnostic evaluations of the pet rabbits in this study. Notably, since it is equipped with software that synchronizes images with respiration, as well as 2 and 3-dimensional (2D/3D) image processing software, the device is extremely easy to use [10-14].

## Results

### Imaging

**Figure 2:**
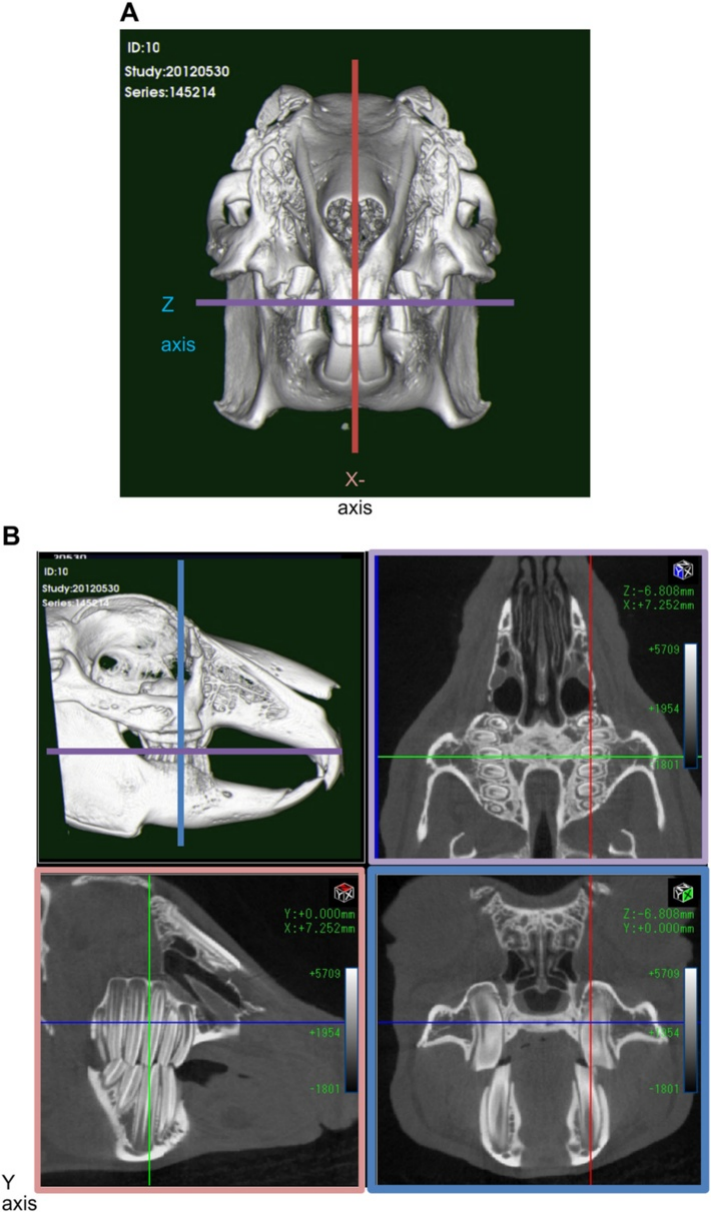
**Planar images of the sagittal, transverse, and dorsal axes, represented as the (A) X, (B) Y, and Z-axes, respectively.** Three-dimensional (3D) images were reconstructed on the basis of two-dimensional (2D) images.

The resolution of the 2D and 3D images acquired allowed for a thorough assessment of each animal’s condition. The structure of the upper and lower jaw, the tooth structure, and the extent of the occlusion between the upper and lower jaws (which is difficult to evaluate by radiography and endoscopy) were visualized clearly, with sufficient detail (Figure 2).

### Evaluation criteria

**Figure 3:**
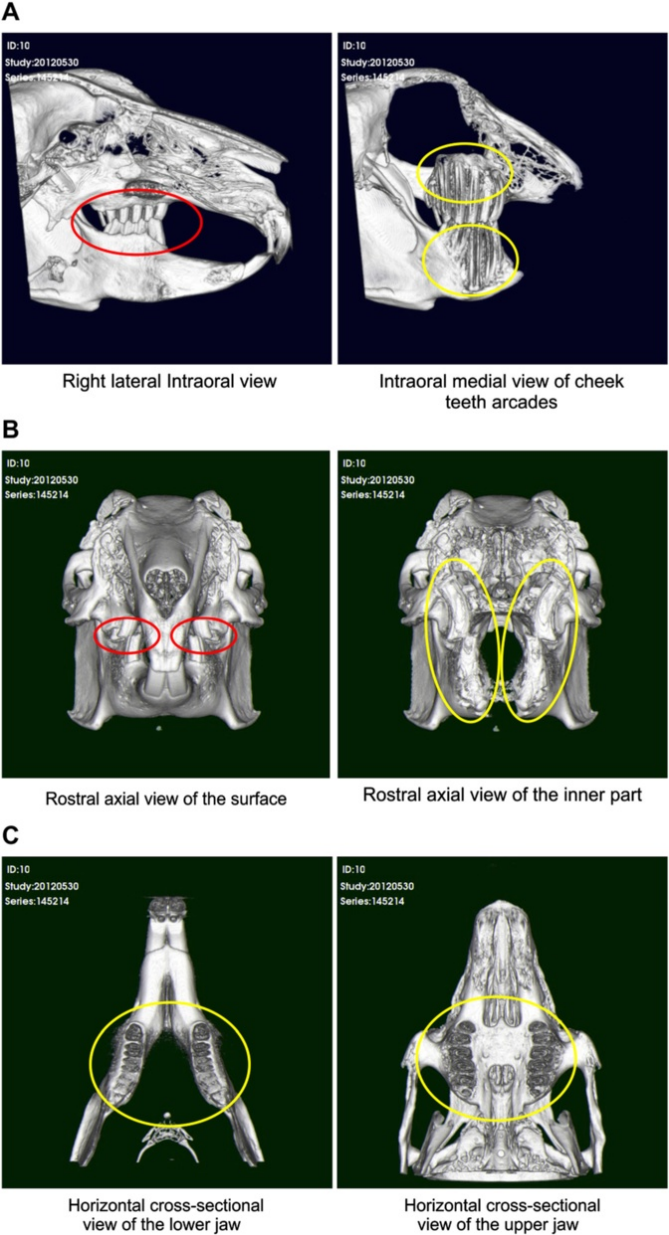
**3D images of the lateral, axial, and horizontal cross-sectional view. (A)** The X-axis shows a right lateral internal view and a cross-sectional view. **(B)** The Y-axis shows rostral axial view at the surface (red circles show the occlusion between the upper and lower premolars and molars) and the rostral axial view of the inner part (yellow circles show the occlusion at the bone level). **(C)** The Z-axis shows a horizontal cross-sectional view of the lower jaw (yellow circle shows the mandibular premolars and molars) and the Horizontal cross-sectional view of the upper jaw (yellow circle shows the maxilla premolars and molars).

As there are no standardized diagnostic criteria, we defined the sagittal plane as the X-axis, the transverse plane as the Y-axis, and the dorsal plane as the Z-axis (Figure 2). The 3D images used in the diagnosis showed right lateral internal views, as well as right lateral internal cross-sectional views. The condition of the apices of the premolars and molars was assessed. The occlusion between the upper and lower jaw incisors, and between the premolars and molars could be easily observed via the X and Y-axes. Dorsal cross-sectional images of the lower and upper jaws were observed from the Z-axis (Figure 3).

The 3D images clearly depicted alterations and deformities in the upper jawbone and teeth, as well as the absence, excessive length, or curvature of premolars and molars, and facilitated determination of the 3D positional relationship.

### Rabbit oral cavity pathology as observed by endoscopy and micro-CT imaging

Endoscopy revealed traumatic ulcers in the buccal mucosa. Accurate diagnosis by endoscopy requires positioning using a suitable device in conjunction with sedation, and is thus associated with an anesthetic risk. In contrast, it is often possible to use micro-CT to capture images without anesthesia.

**Figure 4:**
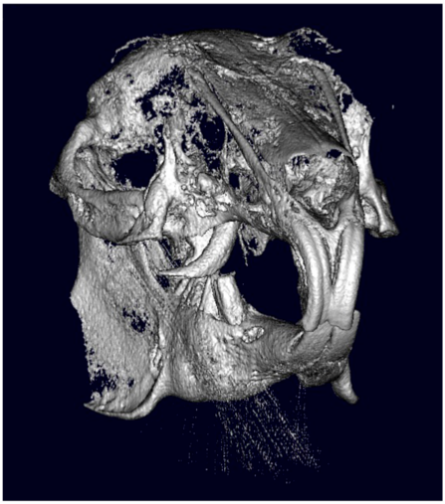
**Computed tomography (CT) image of a diseased oral cavity of a rabbit.** The upper jaw first molar curves outwardly and is excessively long, and the tip has become sharp, leading to damage to the buccal mucosa, and ulcer formation.

**Figure 5:**
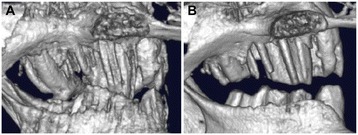
**CT images of elongated premolars and molars, taken before and after treatment. (A)** A case of excessively long premolars and molars. **(B)** Image taken after treatment.

Micro-CT images are an excellent means of acquiring accurate information on changes in the molars of rabbits whose owners are unable to check their appearance macroscopically (Figure 4). Anesthesia can be a major risk in some rabbits. In 90% or more cases, rabbits whose molars have small spikes can be micro-CT imaged effectively without anesthesia, and when the mouth of the rabbits can be opened enough, the molar spikes can be removed with a small clipper. This improves their clinical symptoms. When coronal reduction of molars is properly performed, as opposed to the simple burring of spikes, there is a significant difference in the treatment interval. CT images before and after treatment are shown in Figure 5. The length of symptomatic crowns was reduced, and occlusion between molar arcades was improved. The improvement in clinical symptoms, visual assessment using the laryngoscope, and endoscopic observations confirmed that there was normal healing, and that no occlusal problems were induced.

### Mandibular osteolysis

**Figure 6:**
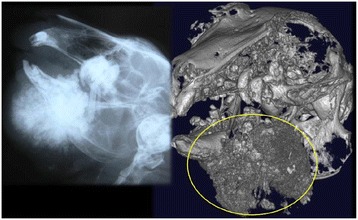
**Comparison between the radiologic findings and a CT image of lower jaw osteolysis.** Radiographic image of mandibular osteolysis; inset, the CT image.

Radiologic findings allowed for the detection of focal lesions in the apical part of molars. Pathological symptoms were also clearly visible on 3D images, such as pus around the dental apices, bone resorption lesions, and mandibular fractures, and provided detailed image information regarding anatomical, dental, and skeletal abnormalities (Figure 6). While 2D CT images are always acquired initially, 3D surface and volume rendering images facilitate the evaluation of locations exhibiting morbid changes.

**Figure 7:**
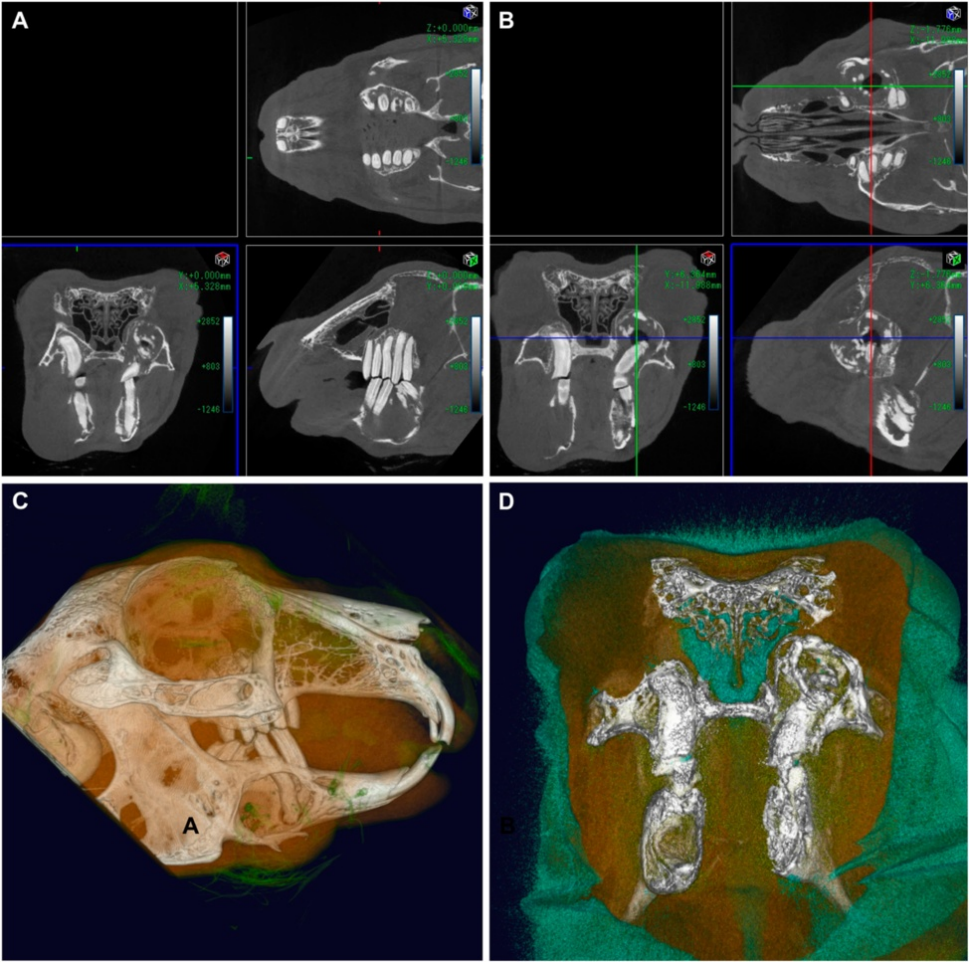
**CT images of osteomyelitis. (A, B)** CT imaging of a 6-year-old male Holland lop rabbit weighing 1.9 kg. Osteomyelitis of the right and left mandible following periapical infection of a premolar tooth is visible. **(C, D)** 3D volume-rendering reconstruction, right lateral view of the same rabbit. The 3D image shows the extent of osteomyelitis of the right mandible **(C)**, while cross-sections **(D)** reveal the extent of osteomyelitis in the left mandibular region.

Bone resorption was thought to indicate periapical infection of the mandibular incisor, or bacterial osteomyelitis which is generally visible in 2D images. 3D images help to determine the extent of osteomyelitis in the region (Figure 7). While 2D CT images are indispensable, 3D surface and volume rendering images make it easier to diagnose pathological changes. In addition, we were able to safely examine all the animals included in the study.

## Discussion

Our findings demonstrated that the micro-CT approach used in this study can be utilized for diagnosis in clinical settings. The equipment facilitated the collection of accurate data, as well as a rapid image processing time and reconstruction time (approximately 1 minute). Because the micro-CT device allows for reconstruction of images derived from slice image data with pixel sizes ranging from 120 μm^2^ to 150 μm^2^, the images are precise, and objects are very clear in comparison to those from conventional CT. The same performance has also been achieved with the imaging of laboratory animals. [10,12,13] While the bone structure and detailed morphological aspects of teeth in rabbits have been difficult to visualize with conventional CT, which is designed for humans, because of the inherently small size of the rabbits’ skull and teeth they are clearly depicted by micro-CT.

In this study, we attempted to compare the diagnostic utility of 2D and 3D images. 2D images allowed for a comparative study of the length, thickness, and curvature angles of teeth via numerical evaluations. These data were obtained by conducting imaging only once, minimizing the animals’ exposure to radiation. However, osteolysis and bone hyperplasia were difficult to identify with 2D imaging, hence we also utilized 3D images. Another advantage of this device is that the images generated are digitally processed in conjunction with every CT scan; consequently, they can be rotated in three dimensions, facilitating an enhanced understanding of the physical presentation of diseases or abnormalities evident in the images.

The excellent imaging capability of micro-CT allowed for the visualization of pathological images associated with diseases specific to rabbits. In the evaluation of each of the individual sites investigated, 2D images enabled the assessment of dentition in the X, Y, and Z-axes on a planar surface, and 3D images enabled the assessment of 3D structure. Thus, the advantages of the micro-CT scanning include the following: (1) Overall assessment of dentition and occlusion, and evaluation of the morphology of the upper and lower jaw, as well as abnormalities in terms of their length and angle (including abnormal angulation against the maxilla/mandible, which can worsen as the teeth continuously grow). (2) Evaluation of deformity, excessive length, and occlusion in the clinical crown. (3) Correlation between the alveolar bone and excessive elongation in the dental apices or deformities of the dental apices. (4) Changes in resorption due to bacterial infection of the dental apex, proliferative changes, fracture-related deformities, and occlusal changes in the upper and lower jaw. Furthermore, the changes identified included dental, skeletal, anatomical, and pathological abnormalities, excessive elongation, and internal and external deviation of the orientations of upper and lower jaw molars, which were difficult to detect by radiography.

The device described in this study allowed for comprehensive assessments via clear imaging of lesions such as those associated with changes in the dental apices and alveolar bones in a variety of disease contexts, including animals with chronic severe diseases. These evaluations can result in more accurate prognoses. The clear image data obtained via micro-CT facilitated the provision of informative explanations to the owners, and was extremely useful with regard to obtaining informed consent for subsequent procedures. Furthermore, the data obtained from the imaging device was useful for educating the owners about the importance of conducting routine monitoring for changes such as a refusal to eat food, spilling of food, reduced amount of stool, drooling, and nasal discharge [2].

According to Brodbelt et al. [15], the incidence rates of anesthetic and sedation-related death in healthy animals were 0.05% in dogs, 0.11% in cats, and 0.73% in rabbits. These increased to 1.33%, 1.40%, and 7.37%, respectively, in diseased animals. With regard to rabbits, it has been found that they usually remain still and calm when a net is placed over them in the manner shown in Figure 1. It was possible to acquire most of the images in this study without anesthesia. It was also possible to apply this method to rabbits with reduced cardiopulmonary function and respiratory function, for which death due to anesthesia was a distinct possibility. Also, the methods used were effective in cases where the owner agreed to CT image diagnosis but not anesthesia. Since the risk of adverse events related to anesthesia and sedation is increased in sick rabbits, it is considered an advantage that images could be acquired without anesthesia.

While dental diseases in rabbits develop gradually, we have diagnosed cases in which a failure to treat the condition during the acute phase caused the animal’s condition to deteriorate severely. To avoid such cases, the diagnosis needs to be made as early as possible. The positioning device described in this study is useful to this end, as it can be used to conduct diagnostic procedures while avoiding the risks associated with the use of anesthetics during endoscopic examination of the rabbit’s deep and narrow oral cavity.

## Conclusions

Micro-CT can provide new and accurate image data that have not been possible to obtain fully with conventional diagnostic methods, and are important for the definitive diagnosis of significant dental and oral lesions in rabbits. We have determined that tests performed using this device can be utilized to accurately diagnose and elucidate the details of dental diseases in rabbits.

### Background section

We describe the diagnostic capabilities of a modified micro-CT scanning approach for the evaluation and treatment planning of oral pathologies, including malocclusion and abscesses, in a sample of 50 rabbits exhibiting symptoms consistent with these abnormalities. Our imaging regimen was streamlined, and minimally invasive as described in our “*Materials and methods*” section. Furthermore, the quality of the scan data we obtained during this investigation permitted differential diagnosis and clinically meaningful resolution of oral malformations that are typically difficult to visualize using methods such as oral endoscopy and X-ray radiography.
